# Probing polarization response of monolayer cell cultures with entangled photon pairs

**DOI:** 10.1002/jbio.202400018

**Published:** 2024-06-21

**Authors:** L. Zhang, V. R. Besaga, P. Rühl, C. Zou, S. H. Heinemann, Y. Wang, F. Setzpfandt

**Affiliations:** ^1^ Institute of Microelectronics of the Chinese Academy of Sciences Beijing China; ^2^ Institute of Applied Physics, Abbe Center of Photonics, Friedrich Schiller University Jena Jena Germany; ^3^ University of the Chinese Academy of Sciences Beijing China; ^4^ Center for Molecular Biomedicine, Department of Biophysics Friedrich Schiller University Jena Jena Germany; ^5^ Jena University Hospital Jena Germany

**Keywords:** entangled photon pairs, monolayer cell cultures, polarization projective measurements, quantum polarization state tomography

## Abstract

This study addresses the critical need for high signal‐to‐noise ratio in optical detection methods for biological sample discrimination under low‐photon‐flux conditions to ensure accuracy without compromising sample integrity. We explore polarization‐based probing, which often excels over intensity modulation when assessing a specimen's morphology. Leveraging non‐classical light sources, our approach capitalizes on sub‐Poissonian photon statistics and quantum correlation‐based measurements. We present a novel, highly sensitive method for probing single‐layer cell cultures using entangled photon pairs. Our approach demonstrates potential for monolayer cell analysis, distinguishing between two types of monolayer cells and their host medium. The experimental results highlight our method's sensitivity, showcasing its potential for biological sample detection using quantum techniques, and paving the way for advanced diagnostic methodologies.
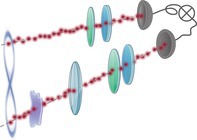

## Graphical abstract

Innovative optical detection using polarization and quantum techniques is presented for biological sample analysis. This method utilizes non‐classical light, specifically entangled photon pairs, for enhanced sensitivity in low‐photon‐flux environments. It excels in distinguishing between cell types and their mediums in monolayer cultures, leveraging sub‐Poissonian photon statistics and quantum correlations. This approach represents a significant advancement in precise, non‐invasive biological sample discrimination, showcasing its potential in advanced diagnostic applications.

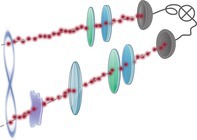

**Abbreviations**
CHO: Chinese hamster ovary
HEK: Human embryonic kidney
LP: Linear polarizer
PBS: Phosphate buffered saline
PSA: Polarization state analyzer
QWP: Quarter‐wave plate
SEM: Standard error of the mean
SNR: Signal‐to‐noise ratio

## 1 INTRODUCTION

Compared to sensing with classical light, non‐classical states of light can enable lower fluctuations in the photon number [1], enhanced signal‐to‐noise ratio (SNR) [2] and thus are suitable for low‐light level sensing and imaging [3]. In particular, non‐classical light can have sub‐Poissonian statistics, where the fluctuation in the photon number is below the classical shot‐noise limit [4]. This reduced fluctuation is particularly advantageous in applications requiring high‐precision sensing, where it leads to improved SNR and thus higher sensitivity.

In addition to fundamentally surpassing classical limits for the SNR using lower fluctuations of the photon number in non‐classical light, typically used measurement techniques for non‐classical states of light, such as correlation measurement, can also lead to an effective reduction of environmental noise caused by detectors or scattered light in the system. For instance, Kalashnikov, et al. [5] compared the SNR in spectroscopic measurements using non‐classical and classical light sources. With their experimental setting, the SNR using a non‐classical light source was 70 000 times greater than that of spectroscopy with classical light.

In low‐light level sensing and imaging, thermal and pseudo‐thermal light sources are not optimal for measurement due to their relatively high intensity fluctuations. For non‐classical light sources, the minimal uncertainty is given by the Heisenberg limit, which reduces the system's measurement uncertainty by a factor of $\sqrt{N}$ compared to the best classical case, where N is photon number involved in interaction with the object under study [1].

Due to the features described above, non‐classical states of light promise to have significant advantages in cell‐culture probing because of benefits from enhanced SNR and a noise reduction in weak‐light measurements that will not damage live cells [6].

Polarimetry, the measurement and analysis of the polarization of light, can also be highly effective in biological sample probing due to its non‐invasive and sensitive nature. It was implemented for diverse application such as microstructural feature characterization [7], probing the retinal network [8], diagnostic early gastric cancer detection [9], studying cell and tissue properties during zebrafish embryonic development [10], evaluating the freshness of ingredients [11], as well as surgical and further diagnostic applications [12]. In all the mentioned studies, classical light sources were used.

In this work, we endeavor to increase sensitivity and precision with enhanced SNR in polarimetric sensing by employing non‐classical states of light. We studied the feasibility of applying polarization‐entangled photon pairs for probing monolayer cultures of mammalian cells with weak polarization response. We experimentally demonstrate the potential of such an approach by distinguishing different cell cultures based on the quantum state changes. Our results promise a practical potential in biological sample characterization.

## 2 EXPERIMENTAL REALIZATION

### 2.1 Cell culture preparation

#### 2.1.1 Cell culture

**FIGURE 1:**
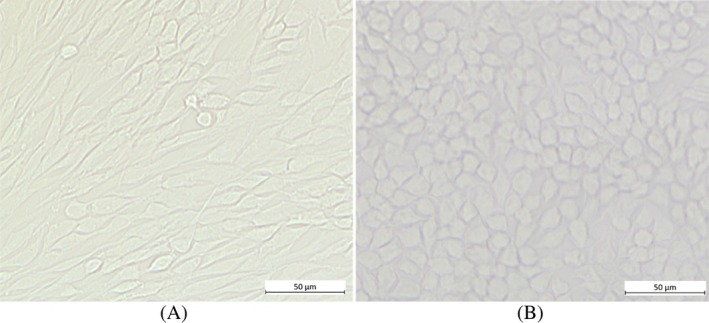
Representative images of cell cultures used in this study. (A) Chinese hamster ovary cells (CHO‐K1), (B) human embryonic kidney cells (HEK293T).

In this study the following mammalian cell lines were used: CHO‐K1 Chinese hamster ovary cells (CHO) were obtained from the German Collection of Microorganisms and Cell Cultures (Braunschweig, Germany) and HEK293T human embryonic kidney cells (HEK) were acquired from the Centre for Applied Microbiology and Research (Porton Down, Salisbury, UK). Cells were cultivated in DMEM/F12 (Thermo Fisher Scientific, Waltham, Massachusetts, USA) and maintained in a humidified incubator with 5% CO_2_ at 37°C. Representative images of cultivated confluent cell cultures (Zeiss, with EC Epiplan‐Neofluar 20×/0.5 HD DIC M27 objective) are shown in Figure 1.

CHO‐K1 and HEK293T were employed because they are well‐characterized cell lines, with well‐established preparation techniques, which avoid potentially unclear effects induced by the samples and ensure samples with spatially homogenous properties. In the reported proof‐of‐principle experiments, employment of the well‐characterized, well‐prepared, homogenous cell lines is crucial to prove the feasibility of the proposed method for further studies and demonstrate its potential in biological sample analysis.

#### 2.1.2 Preparation of fixed cells

Cells were grown on 12‐mm borosilicate glass coverslips (Epredia, Breda, Netherlands) in a 24‐well plate (Greiner Bio‐One, Frickenhausen, Germany) until they formed a confluent monolayer. For fixation, the cell culture medium was removed. The cells were washed three times with 1 mL of phosphate‐buffered saline (PBS, pH 7.4), fixed for 5 min with a 4% paraformaldehyde solution (PFA, pH 6.9) at ambient temperature, and kept in PBS. The fixed samples were rinsed once with deionized H_2_O, and the coverslips were mounted on 76 × 26 mm^2^ glass slides (Thermo Scientific) using Immu‐Mount (Thermo Scientific). For control measurements, samples containing only Immu‐Mount were prepared as control samples, where glass coverslips were washed once with deionized H_2_O and mounted with Immu‐Mount (host material of cells) on a glass slide, to investigate the relative polarization response caused by the CHO and HEK cell monolayers. Control samples are named to be Immu‐Mount with the same logic as cell samples. For each condition, multiple areas from two coverslips were recorded.

### 2.2 Implementation of quantum polarimetric probing setup

**FIGURE 2:**
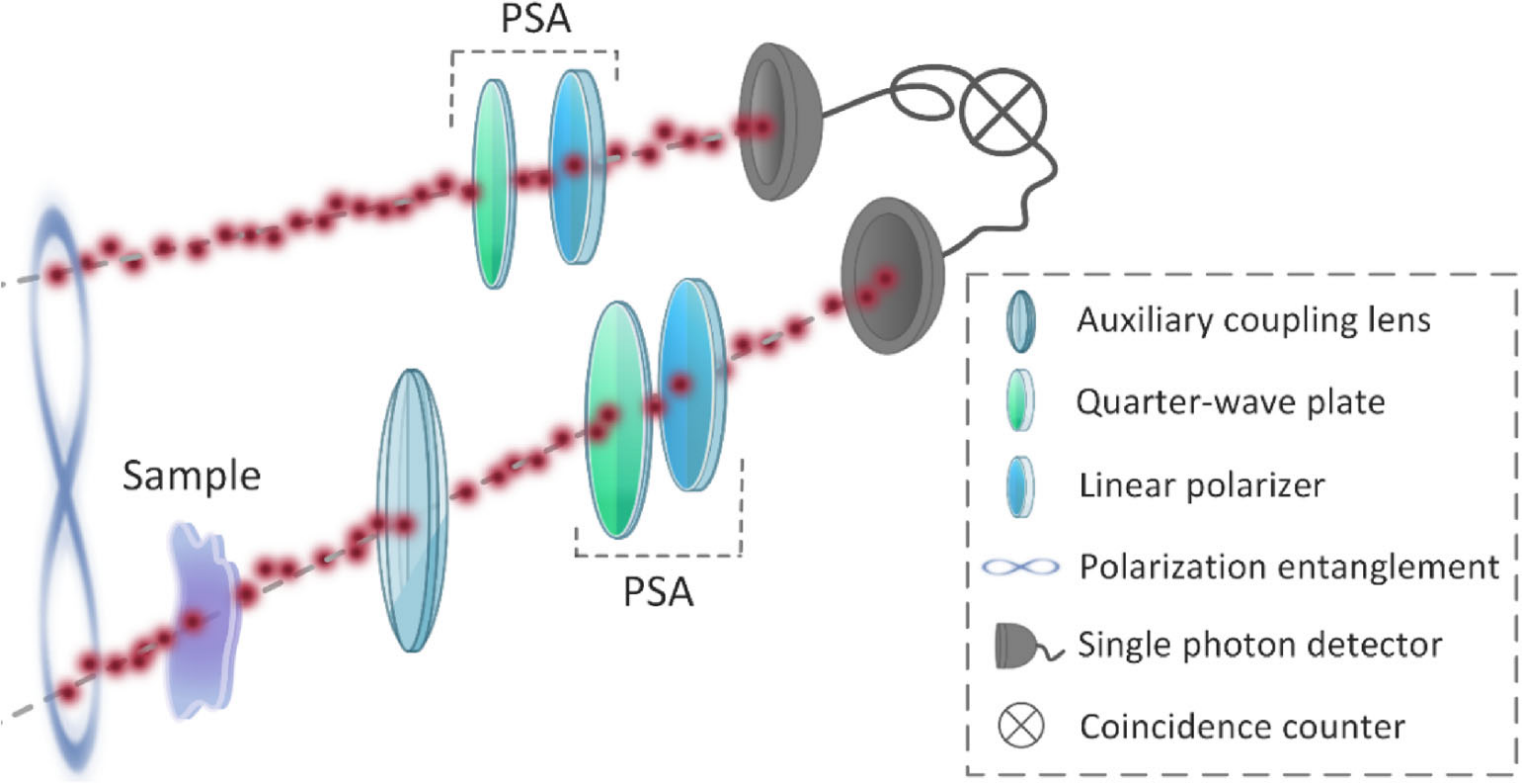
Conceptual sketch of the optical setup used in the experiments. A pair of polarization‐entangled photons is split into two channels. Polarization state analyzers (PSA) that consist of a quarter wave plate and a linear polarizer are introduced in each channel before the detection for polarization projective measurements. A sample under study is introduced into one of the channels (signal channel). A low‐focusing auxiliary coupling lens is introduced after the sample to enhance collection of the photons scattered by the sample towards the detector. The second path acts as a reference channel. Coincidence events between the channels are measured with a time tagging device.

To study the feasibility of the polarimetric probing of monolayer of cell cultures with polarization‐entangled photon pairs we utilized the experimental setup sketched in Figure 2.

The wavelength‐degenerate polarization‐entangled photon pairs at 810 nm are generated with two periodically poled potassium titanyl phosphate (KTiOPO_4_) crystals with type‐II phase‐matching within a polarization Mach‐Zehnder interferometer [13] (not depicted in the figure).

The quantum state of the entangled source used in the study ideally is $\left|\Phi \right\rangle =1/\sqrt{2}\;\left(\left|\mathrm{HV}\right\rangle +\left|\mathrm{VH}\right\rangle \right)$, where H and V denote horizontal and vertical polarization, respectively.

To characterize the state emitted by our source and also the changes induced by the cell samples under test, we employed quantum‐state tomography [14] in the polarization degree of freedom. Here, a polarization state analyzer (PSA) that consists of a quarter‐wave plate (QWP) and a linear polarizer (LP) was used in each channel of the instrument to project the photons into different states. Coincidence events between the two channels were measured using fiber‐coupled single‐photon detectors and a time‐tagging device.

To obtain the complete information about the quantum state, correlations were measured for 16 distinct combinations of polarization projections [14], which are described in Table 1 by the angles of the QWP's fast axis and the transmission axis of the LP with respect to the global vertical orientation in the laboratory. $\left|R\right\rangle$, $\left|L\right\rangle$, $\left|D\right\rangle$ correspond to right circular, left circular and diagonal polarization, respectively. $\left|\mathrm{HV}\right\rangle$ corresponds to the case when a photon with horizontal polarization is projected in the reference channel, and vertical in the signal channel. The rest of the combinations follow similarly.

**TABLE 1:** The 16 distinct combinations of polarization projections used for quantum polarization state tomography. 0° corresponds to global vertical orientation in the laboratory.

No.	Reference Channel	Signal Channel	QWP_R_	LP_R_	QWP_S_	LP_S_
1	$\left|H\right\rangle$	$\left|H\right\rangle$	45°	0°	45°	0°
2	$\left|H\right\rangle$	$\left|V\right\rangle$	45°	0°	0°	0°
3	$\left|V\right\rangle$	$\left|V\right\rangle$	0°	0°	0°	0°
4	$\left|V\right\rangle$	$\left|H\right\rangle$	0°	0°	45°	0°
5	$\left|R\right\rangle$	$\left|H\right\rangle$	22.5°	0°	45°	0°
6	$\left|R\right\rangle$	$\left|V\right\rangle$	22.5°	0°	0°	0°
7	$\left|D\right\rangle$	$\left|V\right\rangle$	22.5°	45°	0°	0°
8	$\left|D\right\rangle$	$\left|H\right\rangle$	22.5°	45°	45°	0°
9	$\left|D\right\rangle$	$\left|R\right\rangle$	22.5°	45°	22.5°	0°
10	$\left|D\right\rangle$	$\left|D\right\rangle$	22.5°	45°	22.5°	45°
11	$\left|R\right\rangle$	$\left|D\right\rangle$	22.5°	0°	22.5°	45°
12	$\left|H\right\rangle$	$\left|D\right\rangle$	45°	0°	22.5°	45°
13	$\left|V\right\rangle$	$\left|D\right\rangle$	0°	0°	22.5°	45°
14	$\left|V\right\rangle$	$\left|L\right\rangle$	0°	0°	22.5°	90°
15	$\left|H\right\rangle$	$\left|L\right\rangle$	45°	0°	22.5°	90°
16	$\left|R\right\rangle$	$\left|L\right\rangle$	22.5°	0°	22.5°	90°

**FIGURE 3:**
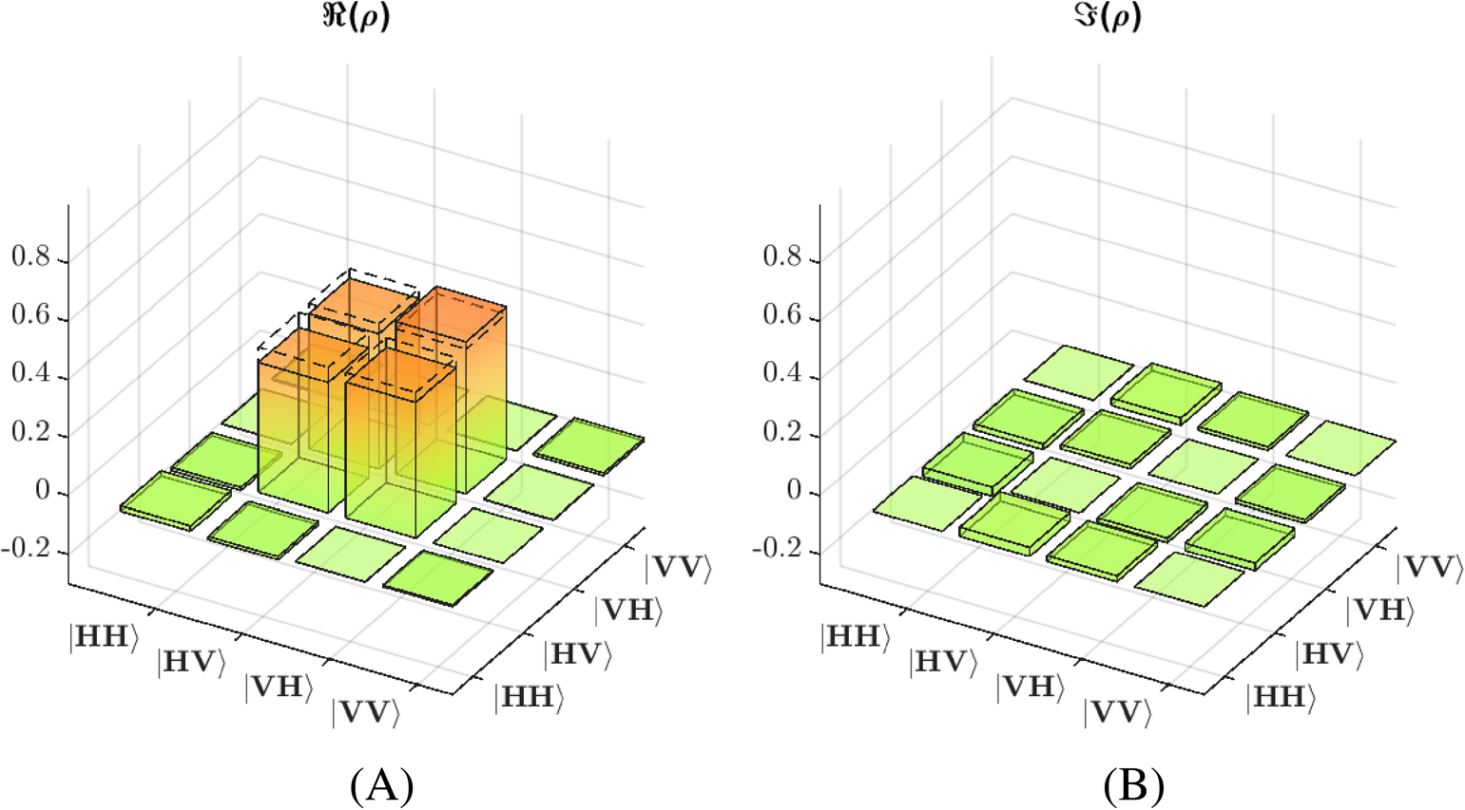
(A) Real and (B) imaginary part of the representative measured density matrix characterizing the quantum state used in the experiments.

First, we characterized the quantum state emitted by our source using quantum tomography. The real and imaginary parts of the density matrix *ρ* calculated from the measured correlations are depicted in Figure 3. The measured density matrix shows a good agreement with the one expected for the state $\left|\Phi \right\rangle$ (marked with dash lines), where the two diagonal elements $\left|\mathrm{HV}\right\rangle \left\langle \mathrm{HV}\right|$ and $\left|\mathrm{VH}\right\rangle \left\langle \mathrm{VH}\right|$, describing the probabilities of measuring the respective combination of photons, have magnitudes close to 0.5. Their mutual coherences in the corresponding off‐diagonal elements also are close to 0.5. Overall, the fidelity of the measured with the ideal quantum state is around 0.95, the slight deviations stems from an asymmetry between the two paths within the source.

The generated photon pairs were divided into two paths so that one of the photons interacts with the sample, while its partner acts as a reference; we denote the first path as signal channel and the other as reference channel.

In the experiments the integral polarization response of the sample is probed, and the sample is illuminated with a collimated beam of approximately 1‐mm diameter. The total number of photons incident on the sample is around 500 000 photons per second, which considering the illumination spot area on the sample, corresponds to photon flux of about 6.4 × 10^11^‐photons/m^2^/s.

No focusing or imaging optics is used before the sample. The integral measurement without spatial resolution governs also the choice of the samples. To examine the sensitivity of the method with respect to sample morphology, different shapes of cell types were selected, and the cell cultures have been prepared as monolayers.

Coincidence counts in all projections after the measured specimen decrease due to polarization‐independent absorption and scattering. To compensate for these losses, we introduced a low‐focusing auxiliary coupling lens after the sample in the signal channel.

To detect the changes in the polarization state of the transmitted photons induced by the cell samples, the quantum state was measured before and after the introduction of samples by obtaining the number of correlations events within 10‐s integration time and 3‐ns coincidence window for the 16 distinct polarizer projection combinations shown in Table 1. The complete measurement procedure for obtaining all necessary data at one sample location is fully automated and takes about 3‐mins.

The obtained coincidence counts were combined into vectors $\psi_{\text{sample}}$ for the measurement with the sample and $\psi_{\text{reference}}$ for the measurement without the sample as:

(2) $$\psi_{\text{sample}}=\left[\begin{array}{c} c_{\mathrm{HH}}^{s}\\ c_{\mathrm{HV}}^{s}\\ \ldots \\ c_{\mathrm{RL}}^{s} \end{array}\right],\;\psi_{\text{reference}}=\left[\begin{array}{c} c_{\mathrm{HH}}^{r}\\ c_{\mathrm{HV}}^{r}\\ \ldots \\ c_{\mathrm{RL}}^{r} \end{array}\right].$$

where c represents coincidence counts and $c_{\mathrm{HH}}^{s}$ are the measured coincidences of the $\left|\mathrm{HH}\right\rangle$ polarizer projection when the sample is measured. In total, 18 samples of CHO, 14 samples of both HEK and Immu‐Mount were measured across both two prepared cell culture areas. For each type of sample, even though the measurements were taken at different times with different samples, the measurement variation of all obtained data lies within 3σ from its average.

### 2.3 Data analysis

The changes in the coincidence counts (here referred to as “coincidence”), which are caused by the presence of the samples, across the 16 distinct polarizer projection combinations was calculated as:

(3) $$\psi_{\text{change}}=\psi_{\text{sample}}-\psi_{\text{reference}}$$

The averaged coincidence and standard error of the mean (SEM) for the measurements were calculated.

The data distribution of the measurements was visualized in box plots. The box area illustrates that 50% of the data points fall within the marked range, the bottom and the top of the box represent the 25th and 75th percentile of the data, respectively.

Wilcoxon rank‐sum test, also known as the Mann–Whitney U test [15], was employed to evaluate whether the measured cell types can be distinguished from each other, where the test is used to determine if there are statistically significant differences between two independent groups. As a measure, we used the p value, where a value below 0.05 indicates that the two compared groups are distinguishable [16]. The Wilcoxon rank‐sum test was chosen for its ability to robustly compare two independent groups in this proof‐of‐principle phase of the method development.

A 4 × 4 matrix representing the impact of the sample under study, further referred to as a change matrix M with elements $m_{\mathrm{ij}}$, was calculated for all cell cultures and the host medium using method employed to calculate the density matrix in quantum polarization state tomography [14]. For this, the values of the vector $\psi_{\text{change}}$ were used as inputs for tomographic reconstruction algorithm including maximum likelihood estimation [15]. The matrix depicts the alteration in polarization of the photons transmitted by the sample.

## 3 RESULTS AND DISCUSSION

When a sample containing either the cell culture grown as a monolayer or a control sample with host material only is introduced into the optical path of photons in the signal channel, different phenomena of light‐matter interaction take place concurrently. In order to address the effects of our cell samples on the polarization of the incident photons, the cell sample response is analyzed relative to the control sample, that is, the host medium Immu‐Mount. If the suggested approach of probing with polarization‐entangled photon pairs is sensitive enough, the response from the monolayer cell samples should be distinct from the response for the host medium alone. Furthermore, if this response is related to specific properties of the different cells, for example, the sample morphology, the polarization response should also be different for the two investigated cell types, whose cells have different shapes and the monolayers having different textures. In the following, we address these questions with statistical tests on the measured data.

### 3.1 Averaged coincidence and SEM of the 16 polarization projections

First, we analyze the outcome from simple polarization projective measurements. To this end, the averaged coincidences and the SEM for all measurements were calculated for each of the 16 distinct combinations of polarization projections shown in Table 1.

**FIGURE 4:**
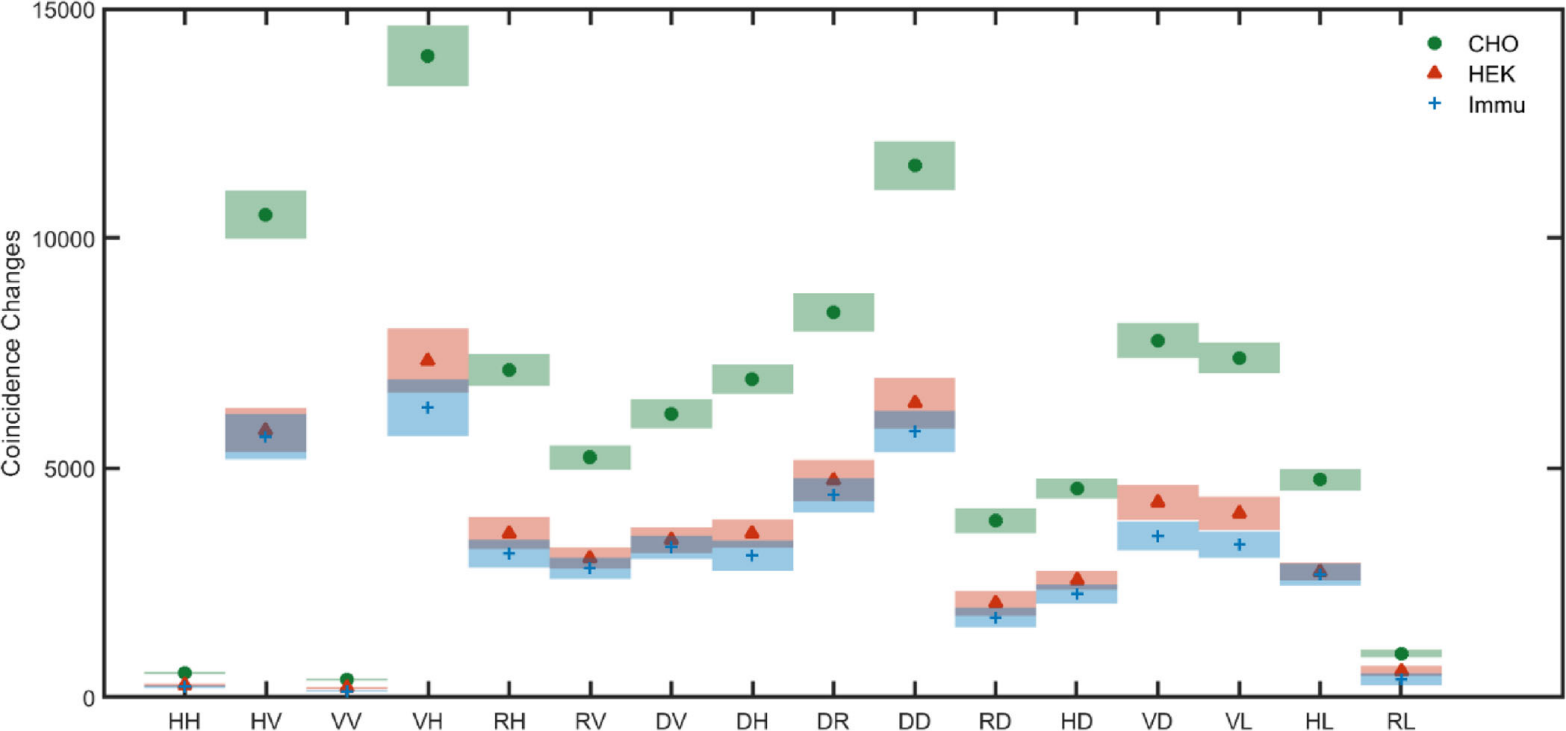
The averaged change in the coincidence counts and standard error of the mean for polarization projective measurements.

The results are displayed in Figure 4, where the labels on the X axis correspond to the polarization combinations as described in Table 1. In the figure, the measurements for all 18 CHO,14 HEK, and 14 Immu‐Mount areas under study contributed to the averaged coincidences changes caused from sample present and SEM. The solid markers represent averaged coincidence and same color shade areas represent corresponding values of SEM.

The values of averaged coincidence and SEM are used in the following for testing whether the sample presence can be detected and whether different cell types can be differentiated from each other.

### 3.2 Discrimination of prepared cell types and host medium

Wilcoxon rank‐sum test was performed for all 18 samples of CHO, 14 samples of HEK, and 14 samples of Immu‐Mount using the values of coincidences $\psi_{\text{change}}$. The p values from Wilcoxon rank‐sum test are displayed in Table 2, in which p values between CHO and Immu‐Mount, between CHO and HEK, and between HEK and Immu‐Mount are contained in the second, third, and fourth columns, respectively.

**TABLE 2:** Wilcoxon rank‐sum test *p* values for 16 combinations of polarization projective measurements.

Projections	CHO & Immu‐Mount	CHO & HEK	HEK & Immu‐mount
HH	6.17 × 10^−6^	4.11 × 10^−5^	6.08 × 10^−1^
HV	7.01 × 10^−5^	4.92 × 10^−5^	1
VV	9.13 × 10^−6^	5.86 × 10^−5^	1.11 × 10^−1^
VH	9.15 × 10^−6^	2.85 × 10^−5^	3.30 × 10^−1^
RH	7.53 × 10^−6^	2.85 × 10^−5^	3.83 × 10^−1^
RV	7.01 × 10^−5^	1.95 × 10^−4^	5.38 × 10^−1^
DV	5.88 × 10^−5^	1.40 × 10^−4^	5.38 × 10^−1^
DH	9.15 × 10^−6^	1.96 × 10^−5^	3.56 × 10^−1^
DR	3.42 × 10^−5^	9.93 × 10^−5^	8.37 × 10^−1^
DD	1.34 × 10^−5^	3.42 × 10^−5^	3.30 × 10^−1^
RD	3.42 × 10^−5^	1.95 × 10^−4^	5.05 × 10^−1^
HD	1.35 × 10^−5^	3.42 × 10^−5^	3.05 × 10^−1^
VD	6.18 × 10^−6^	4.92 × 10^−5^	2.00 × 10^−1^
VL	5.07 × 10^−6^	3.42 × 10^−5^	2.00 × 10^−1^
HL	9.93 × 10^−5^	8.35 × 10^−5^	9.18 × 10^−1^
RL	3.75 × 10^−4^	1.20 × 10^−2^	3.56 × 10^−1^

From the second column, one can clearly see that all 16 distinct combinations of polarization projections differ significantly (*p* < 0.05 for all) between CHO cells and the mounting medium, which means there are significant differences between these two sample types. This indicates the potential of distinguishing CHO cells from the homogeneous host medium using polarization measurements.

To study whether the detected polarization response is explained by solely scattering of photons on the cells or indeed by the polarization selectivity in the transmission due to the morphology of the sample, we investigate whether presence of another cell type (HEK) can be also detected and whether the method shows sensitivity to different cell types.

Therefore, the test was performed between monolayer CHO and HEK cell cultures, and the third column of Table 2 shows that the p values are all far below 0.05, which indicates the potential to distinguish these two cell types.

At the same time, this does not hold true for the testing results between HEK and Immu‐Mount samples, seen in fourth column, which indicates that only employing coincidences of the 16 distinct combinations of polarization projections cannot distinguish HEK cells from the host medium and further methods need to be employed. Therefore, we performed tomographic reconstructions for all the three cell types with the 16 distinct combinations of polarization projections to examine whether the difference between HEK and Immu‐Mount can be extracted from elements of the change matrix M.

### 3.3 Change matrixes of all samples

**FIGURE 5:**
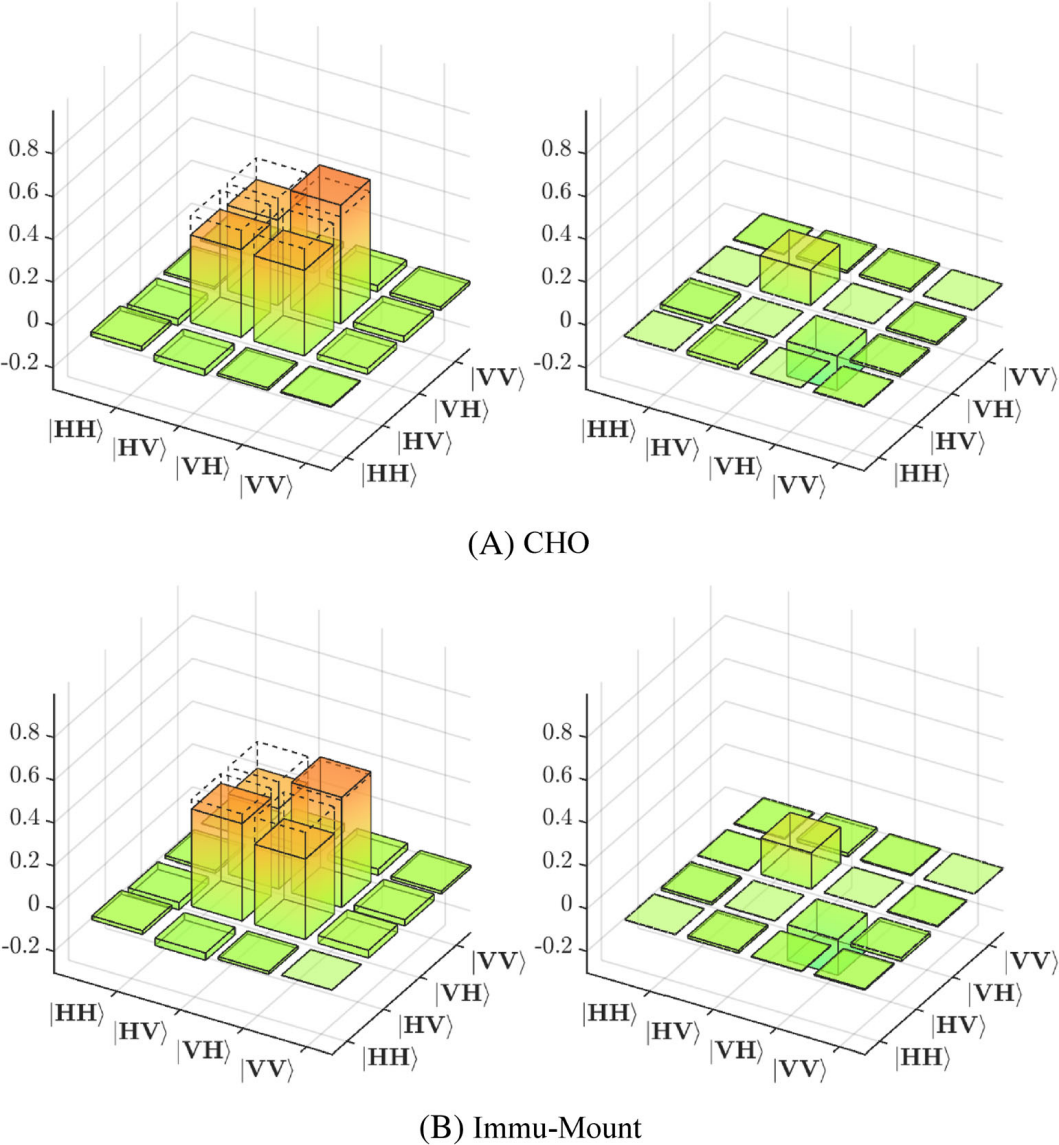
Real (left) and imaginary (right) part of the change matrix of (A) CHO and (B) Immu‐Mount.

The averaged change matrixes of all samples of CHO, and Immu‐Mount are shown in Figure 5 as an example. The SEM of each averaged diagonal element is negligible and therefore is not shown in the figure. Dashed lines in the matrixes display the value 0.5 as a reference to compare. The left column displays the real part of the change matrix while the right column displays its imaginary part.

In order to compare the three types of cell cultures, related elements in the change matrix M were picked out for further analysis. Because of the polarization quantum state of the utilized entangled source, most counts and also maximal changes are expected for the four elements: $m_{2,2}$, $m_{2,3}$, $m_{3,2}$, $m_{3,3}$, where the index number 2 corresponds to $\left|\mathrm{HV}\right\rangle$ and index 3 to $\left|\mathrm{VH}\right\rangle$, respectively. These are the elements containing the amplitudes for $\left|\mathrm{HV}\right\rangle \left\langle \mathrm{HV}\right|$, $\left|\mathrm{HV}\right\rangle \left\langle \mathrm{VH}\right|$, $\left|\mathrm{VH}\right\rangle \left\langle \mathrm{HV}\right|$, $\left|\mathrm{VH}\right\rangle \left\langle \mathrm{VH}\right|$, respectively as shown in Figure 5. Only these four elements were considered for subsequent analysis.

**FIGURE 6:**
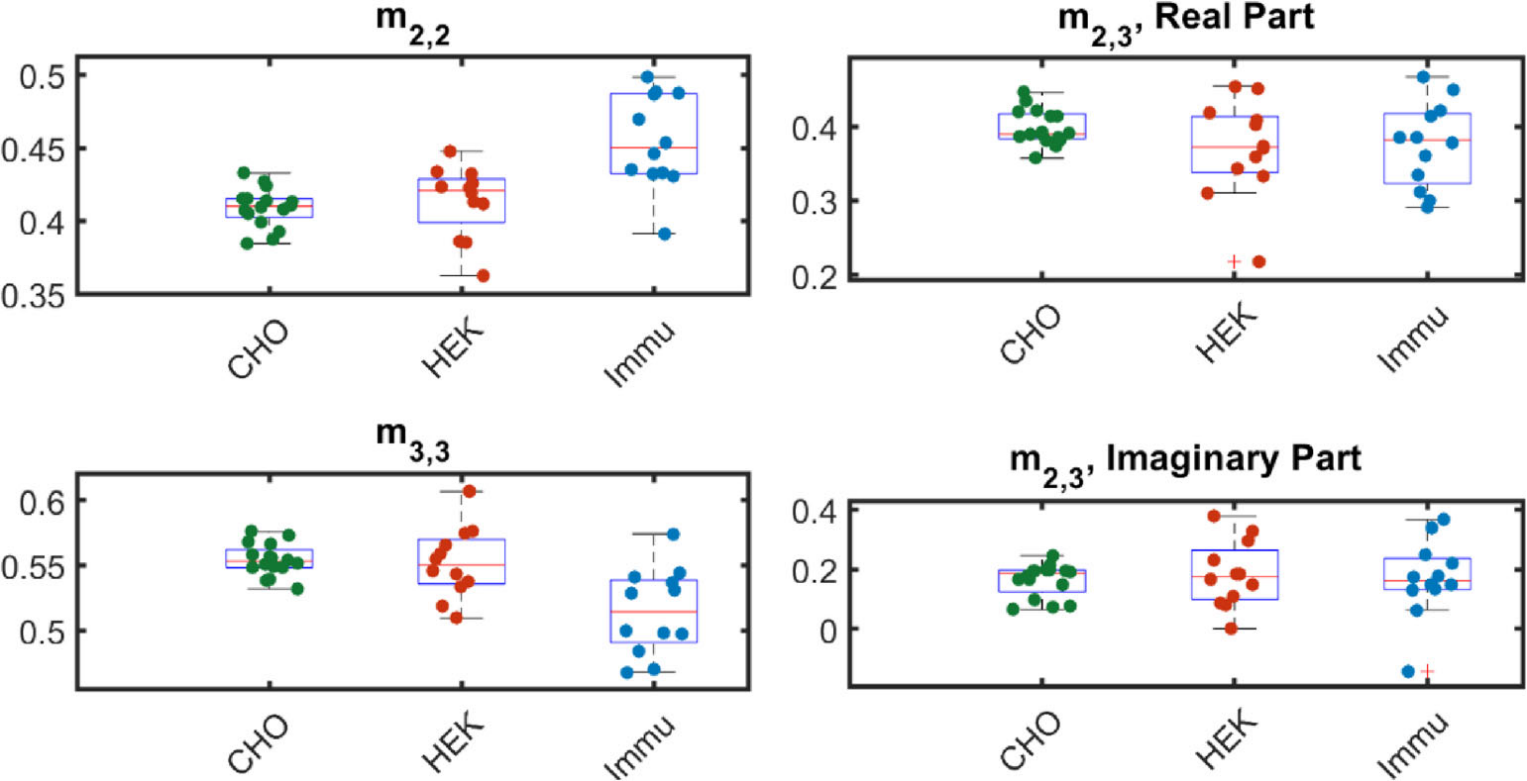
Box plot of elements m_2,2_, m_2,3_ (real and imaginary parts separately), and m_3,3_ of all investigated types of samples: CHO, HEK, and Immu‐Mount.

Box plots of the four elements are shown in Figure 6. Due to the normalization during tomographic reconstruction, $m_{2,3}$, $m_{3,2}$ are complex conjugates of each other. It is therefore sufficient to analyze only $m_{2,3}$, which is analyzed and plotted separately with its real part and imaginary part.

Figure 6 displays the repeatability of elements m_2,2_, m_2,3_‐real, m_3,3_, and m_2,3_‐imaginary parts separately of all investigated types of samples. In order to further differentiate cell cultures from the host medium, we utilized data of m_2,2_ and m_3,3_ for further Wilcoxon rank‐sum test. Due to normalization, the sum of the elements m_2,2_ and m_3,3_ is 1, we picked the data of m_3,3_ for all samples and received the p value of CHO with Immu‐Mount and HEK with Immu‐Mount, which can be seen in Table 5.

**TABLE 5:** Wilcoxon Rank‐Sum Test P Values for CHO and HEK with Immu‐Mount.

Sample types	CHO	HEK
Immu‐Mount	2.24 × 10^−4^	0.01

With the data of m_3,3_ for all samples, the *p* value of HEK with Immu‐Mount diminished to 0.01, which is considered to signify a notable difference between the two species. Here, we successfully differentiate CHO, Immu‐Mount and HEK one from all the others. This indicates that the measured polarization response is related to the morphology of the cells in the studied monolayer cultures. With planned experiments on different density of cells in the samples and extended list of cell types, the presented results have a great potential for highly sensitive detection and differentiation of delicate samples like monolayer cell cultures.

## 4 CONCLUSION

Here we utilized polarization‐entangled photons pairs to assess the potential of quantum polarimetry as a means to identify different mammalian cell types arranged in monolayers. We experimentally demonstrated the sensitivity of this method by distinguishing the response of a cell culture sample from the response of the homogenous host medium. In addition, we could measure differences in the response of two types of monolayer cell cultures, Chinese hamster ovary and human embryonic kidney cells as well as their host material. This holds significant potential, as it is, to our knowledge, the first experiment that has the potential to enable monolayer cell culture differentiation using a polarimetry‐based method.

By comparing polarimetry on biological specimen with classical states of light to our first results using non‐classical light, the potential of using non‐classical light for the investigation of biological samples becomes apparent. For example, the photon flux utilized for cancerous and turbid tissue diagnosis with circularly polarized light [17, 18] was much higher than the photon flux in this study. A lower photon flux while measuring the same information is beneficial, as it ensures the safety of the investigated biological matter. Using synthetic test samples, in a separate study it was demonstrated that nonlocal polarimetry with non‐classical states leads to improved precision and accuracy in extracting information about sample characteristics compared to local polarimetry with classical states of light using the same intensity [19], which means that a similar precision can be obtained with lower intensity using non‐classical light.

Our findings not only contribute to the advancement of biological sample detection techniques but also open new avenues for employing quantum‐assisted methodologies in complex biological system analysis. The method has the advantage of using a very low photon flux to minimize photodamage, which benefits live cell probing, while still providing a high sensitivity. It should be suitable for live cells due to its 3‐min full measurement process, during which cell distribution remains relatively stable. Additionally, the method's effectiveness in distinguishing between cell types is unaffected by the host medium, which produce consistent signal detection across all samples.

One key limitation of the presented approach is the very limited spatial resolution, which limits the presented measurements to homogeneous films of cells. Further research already initiated to realize the spatially resolved tomography. Furthermore, current method is developed to discriminate different cells and will not detect variations in cell density or concentration. This property arises because differences in cell density uniformly affect all 16 projective measurements, causing the changes in the correlation counts to scale linearly with cell density but without altering their proportional relationships within the 16 projective measurements. Due to the normalization of the change matrix, similar change matrices will be obtained for different density of the same cells.

Further experiments fully using the advantageous features of correlation‐based measurement techniques based on photon pairs, such as enhanced SNR, can further extend the limits of this measurement approach.

## AUTHOR CONTRIBUTIONS

L. Zhang conducted the experiments, collected and analyzed the data, designed the algorithms wrote the manuscript, V. R. Besaga conceived the study, designed and implemented the experiments, analyzed the data, edited the manuscript. P. Rühl prepared the samples, analyzed the data, edited the manuscript. C. Zou analyzed the data and edited the manuscript. S. H. Heinemann, Y. Wang, and F. Setzpfandt supervised the research, edited the manuscript. All authors reviewed the manuscript.

## CONFLICT OF INTEREST STATEMENT

The authors declare no financial or commercial conflict of interest.

## Data Availability

The data that support the findings of this study are available on request from the corresponding author. The data are not publicly available due to privacy or ethical restrictions.
